# Supplementary material for: Genetic and structural insights into the functional importance of the conserved gly-met-rich C-terminal tails in bacterial chaperonins
Source: Commun Biol. 2025 Apr 8;8:555. doi: 10.1038/s42003-025-07927-x (PMC11978752; doi:10.1038/s42003-025-07927-x)

Supplementary Information for

Genetic and Structural insights into the Functional Importance of the Conserved Gly-Met-Rich C-Terminal Tails in Bacterial Chaperonins

C. M. Santosh Kumar[1]*, Aisha M. Mai[1], Shekhar C. Mande[2] and Peter A. Lund[1]

[1]Institute of Microbiology and Infection, School of Biosciences, University of Birmingham, Birmingham, UK. B15 2TT

Address for Correspondence:

C M Santosh Kumar: s.k.cm@bham.ac.uk

Sub-title: Function for Carboxy-terminal Segments in Chaperonins.

Key words: GroEL, Carboxy-termini, Conditional Mutants, Normal Mode Analysis, Label-Free Proteomics.

Supplementary Figures and legends

Supplementary figure 1. Phylogenetic tree of the Actinobacterial Chaperonins. This is the rectangular tree version of the tree shown in Figure 1a. Evolutionary analyses of the 325 Actinobacterial chaperonins were conducted in MEGA11 and the phylogenetic relations were inferred using neighbour-joining algorithm. The branches are color coded as in Figure 1a and labelled with the UniProt names of the chaperonins. The numbers on the nodes denote node ages. **The tree is presented at the end of this document.**

```
>EMBOSS0001
MAKIIAFDEEARRGLERGVNxLADAVKVTLGPKGRNVVLEKKWGAPTITNDGVTIAKEIE
LEDPYExIGAELVKEVAxKTNDVAGDGTTTATVLAQALVREGLRNVAAGANPMALKRGIE
KAVEAVTExLLxSAKEVETKEQIAAxATISAxDxxIGELIAEAMDKVGKDGVITVEESNT
xGLELELTEGMRFDKGYISxYFVTDxERQEAVLEDPYILLxxxKISSVKDLLPLLEKVMQ
SGKPLLIIAEDVEGEALATLVVNxIRxTxKSVAVKAPGFGDRRKAMLxDIAILTGGQVIS
EEVGLxLExxxLDLLGxARKVVVTKDETTIVDGAGxxDxIxxRVxQIRxEIExSDSDWDR
EKLQERLAKLAGGVAVIKVGAATEVELKERKHRIEDAVxxAKAAVEEGIVAGGGxALIQA
AxxxxExxxxxxDExxxxxVVxxxxxxxxxxxxxxxxxxGxVVVxxxxxxxxxxxxxAAx
xxxxxLLxxxVIxxxxxxxSAxxxAASAAxxLLTTxxVxxExxExxxxxxxxxxxxxxxx
xxxxxxxxxxxxxxxxxxxxxxxxxxxxxxxxxxxxxxxxxx
```

Supplementary figure 2. Conserved sequence from the Multiple sequence alignment of the chaperonin. The multiple sequence alignment was input into the EMBOSS Cons and the conserved sequences were obtained using default parameters.

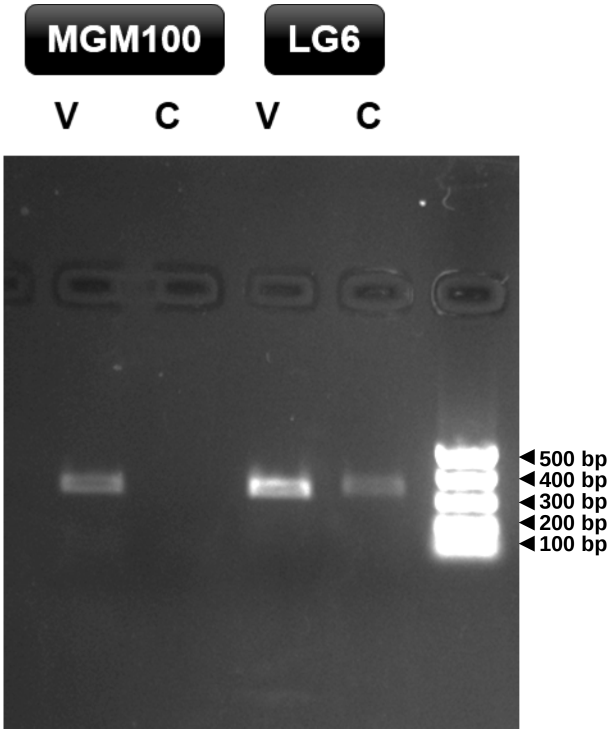

Supplementary figure 3. Reverse transcriptase PCR to detect *groEL* variant expression. MGM100 and LG6 harboring pTrc/GSL and pBAD/GSL, were cultured in the presence of D-lactose and L-arabinose, respectively, to induce the vector-borne *groEL* genes. Levels of expression of chromosome-borne (C) and vector-borne (V) *groEL* genes in the indicated GroE conditional mutant strains were estimated with reverse transcriptase PCR using specific oligonucleotide primers and the resulting products were resolved through a 1.3% agarose gel. The numbers denote the molecular mass standard band sizes.

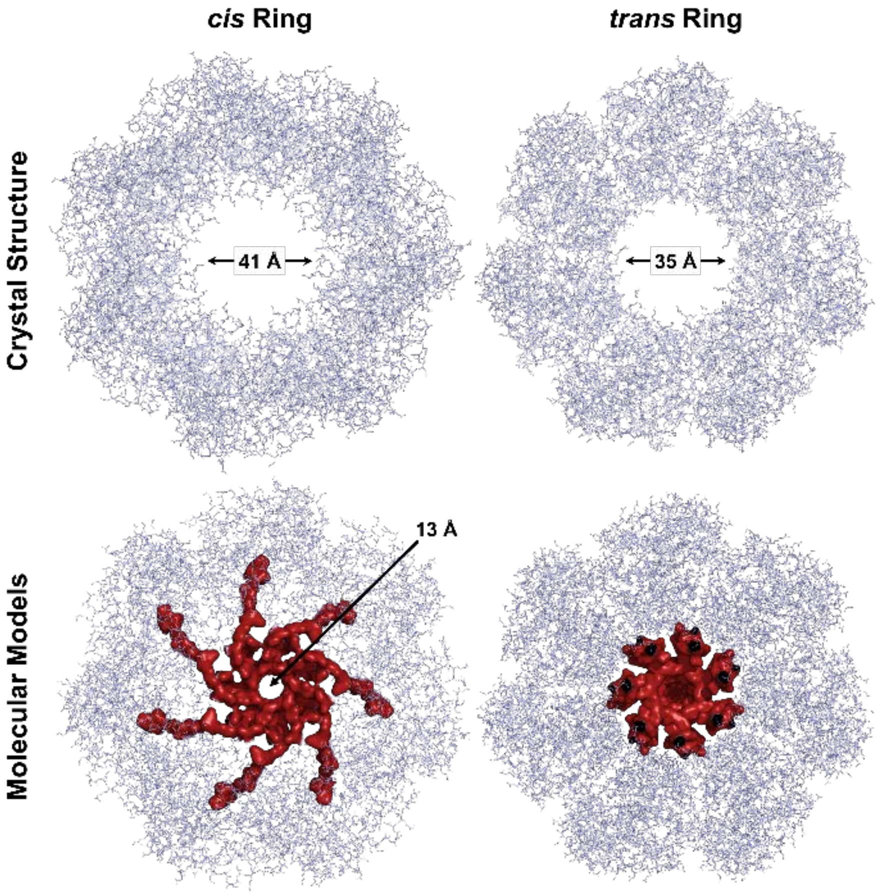

Supplementary figure 4. CTS fills the void at the base of the GroEL cavity. Structural comparison of bottom views of *cis* and *trans* ring models from crystal structure (PDB ID: 1AON), showing the filling up of the void by the carboxy termini segments (CTS) that are shown as space-filled structures in red. Numbers represent the diameter of the void at the bottom of the corresponding rings.

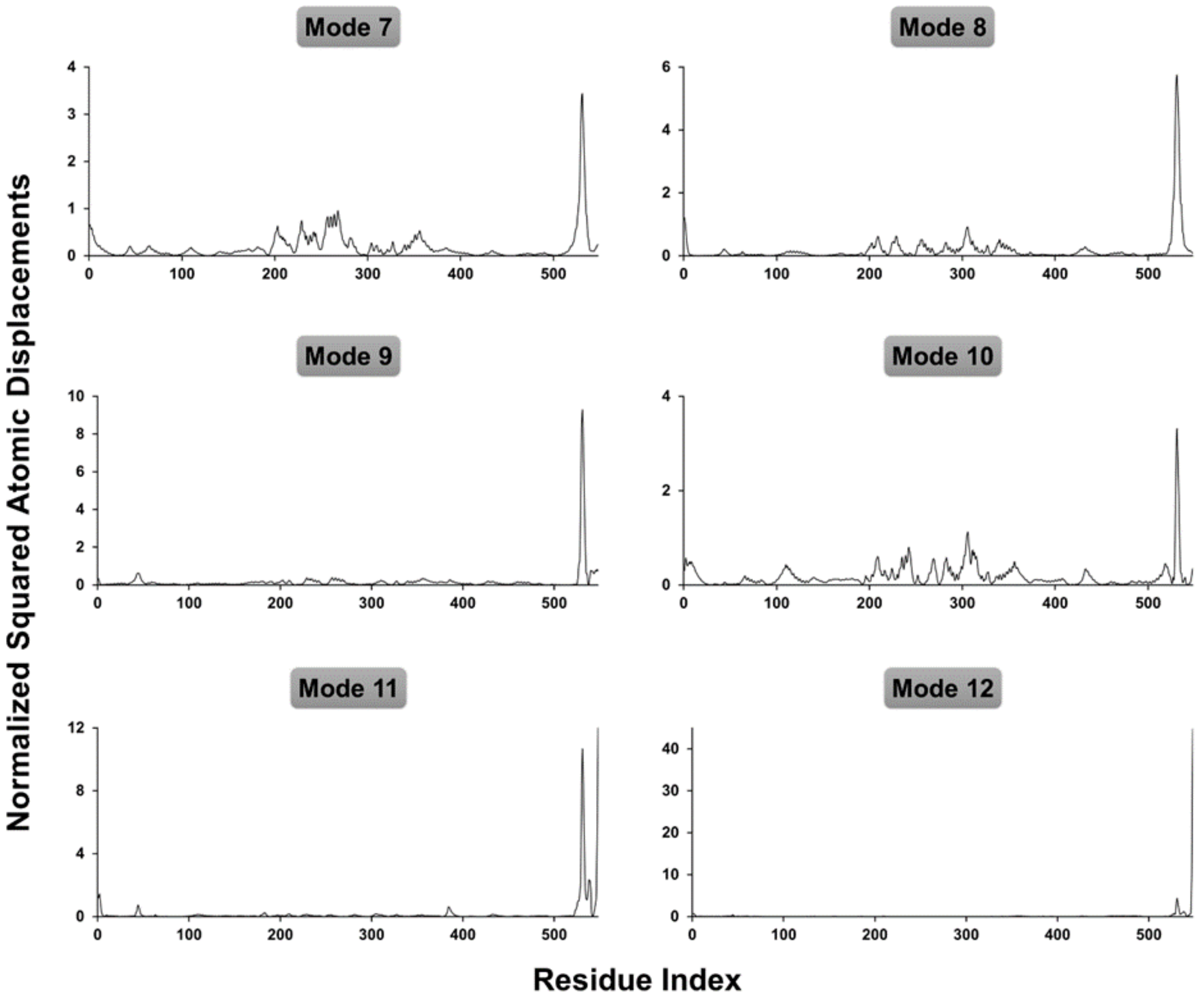

Supplementary figure 5. Fluctuation plots for Normal Modes of GroEL protomer. Residue level fluctuations in the indicated modes for the GroEL protomer are presented.

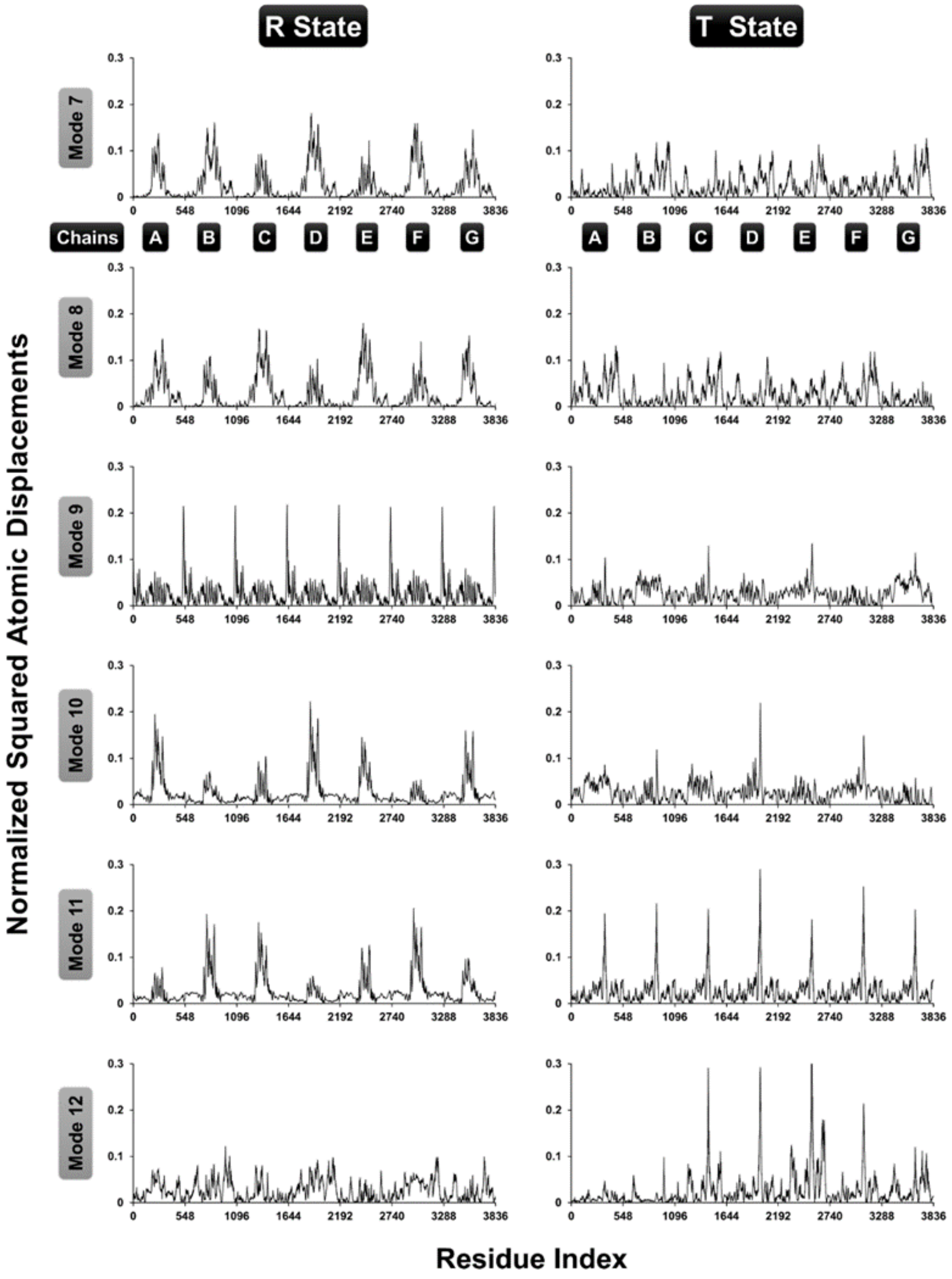

Supplementary figure 6. Fluctuation plots for Normal Modes in GroEL rings. Residue level fluctuations in the indicated modes for the GroEL rings with the indicated conformational states were deciphered by Normal Mode Analysis. The letters A-G represent the positions of the individual chains in the GroEL rings.

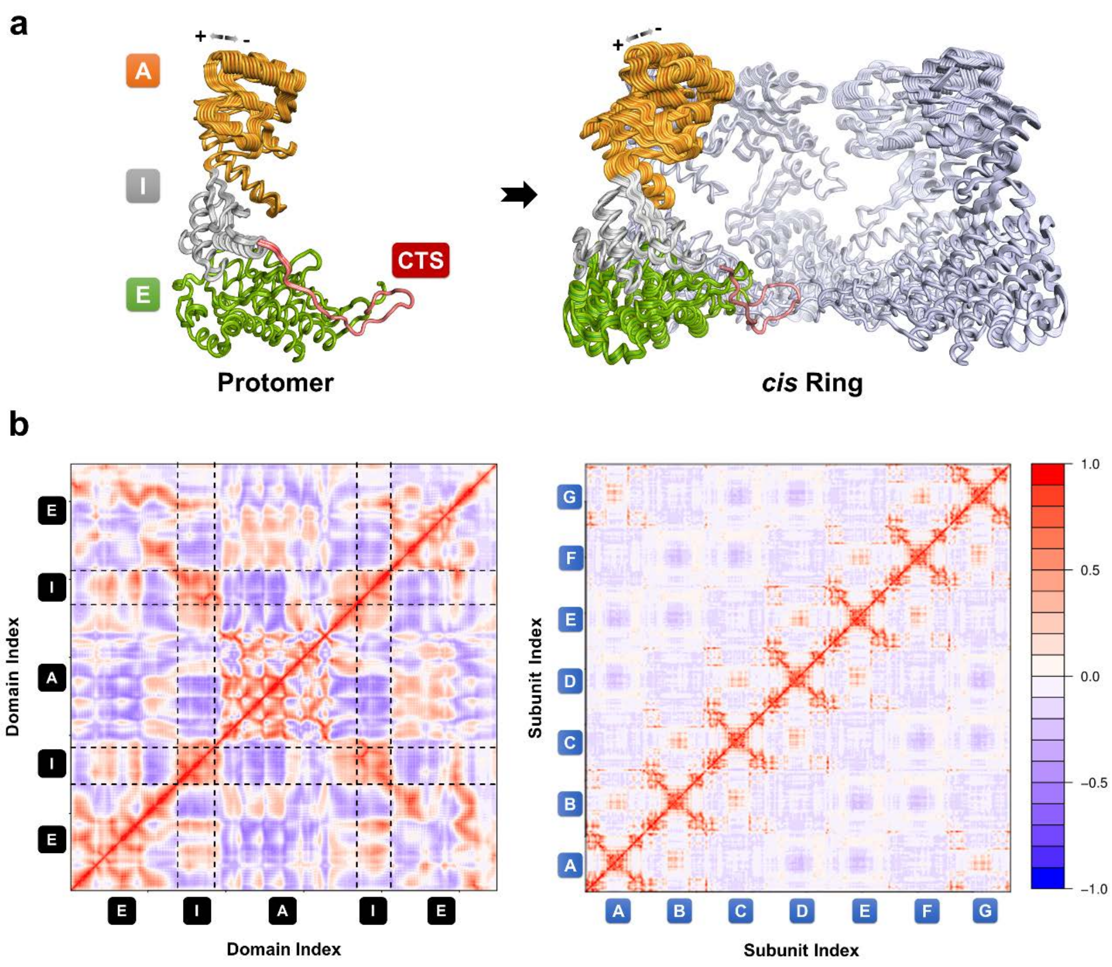

Supplementary figure 7. Normal Mode Analysis shows how cavity size may constrain the flexible GroEL carboxy terminus. (a) Structural displacements of the GroEL protomer and the *cis* ring as predicted by mode 7 in NMA. The dark to light color shades denotes the successive states (V and X lowest non-zero global modes) as they deviate from the input structures (dark shade), in both directions (+ or -) along the reaction coordinates. The domains in the protomer and one monomer in the *cis* ring are color coded as in figure 3. (**b)** Correlation matrices for the protomeric and heptameric GroEL molecules showing differential displacement of the alpha carbon atoms in the indicated domains of the protomer and subunits of the *cis* ring.

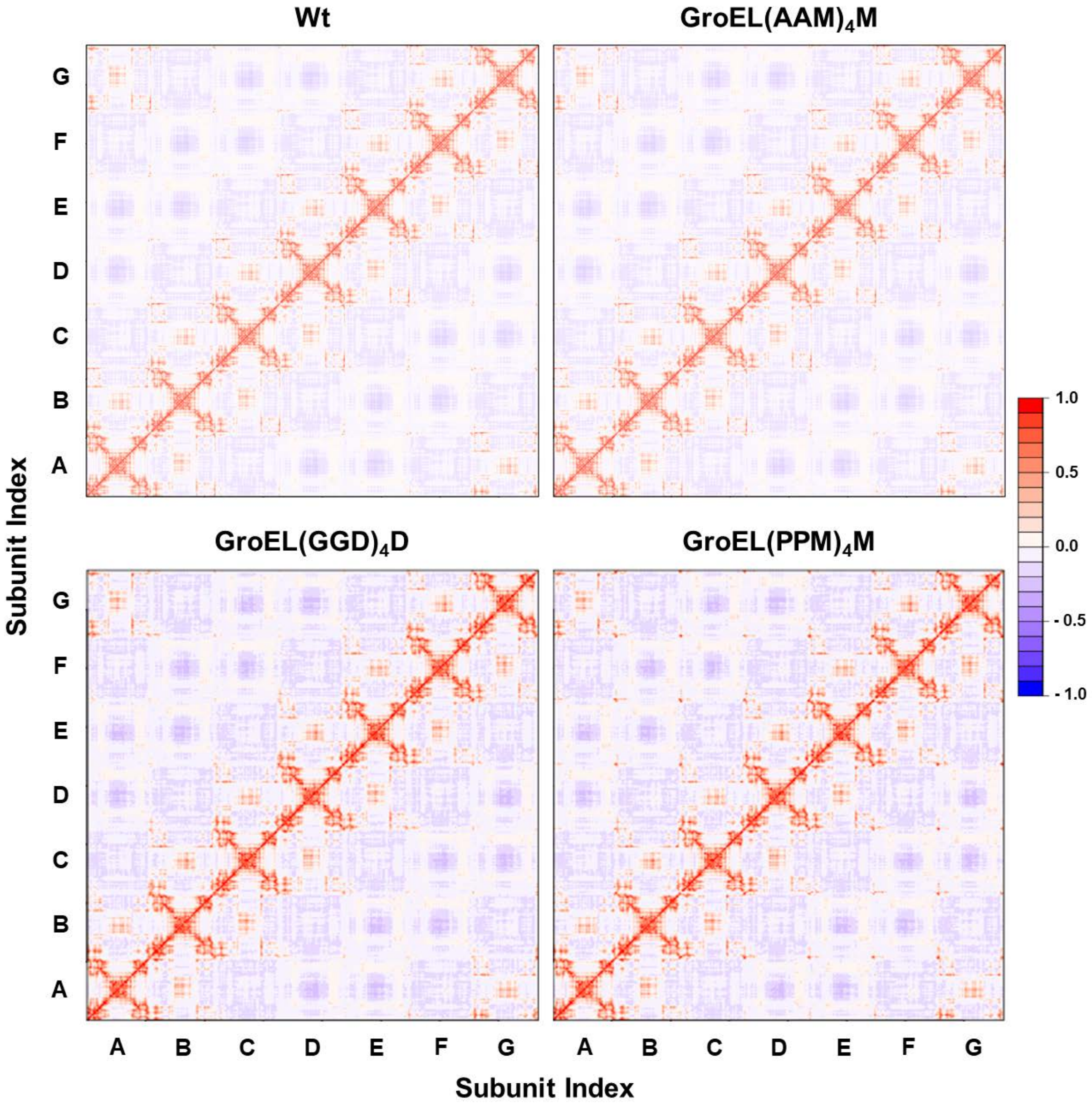

Supplementary figure 8. Correlation matrices of the GroEL oligomers with different tails. Correlation matrices for the heptameric rings of the indicated tail variant GroEL molecules showing domain and subunit displacements. The matrix for the Wt GroEL is reproduced from Supplementary fig. 5b, for comparison.

Supplementary table 1. Oligonucleotide Primers used in this Study.

Name	Sequence	Source or Reference
pBAD F	5′ CTGTTTCTCCATACCCGTT 3′	[1]
pBAD R	5′ CTCATCCGCCAAAACAG 3′	[1]
pTrc F	5′ TGCAGGTCGTAAATCACTGC 3′	[2]
pTrc R	5′ CTGGCAGTTCCCTACTCTCG 3′	[2]
GroELΔC_{13} Stop F	5′ GCAGCTGACTSAGGCGCTSAAGGCGGTATGGGCGGCATG 3′	This study
GroELΔC_{13} Stop R	5′ CATGCCGCCCATACCGCCTSAAGCGCCTAAGTCAGCTGC 3′	This study
GroELΔC_{16} Stop F	5′ CGAAAAACGATGCGGCTGACTAAGGCGCTGCTGGCGGTAT 3′	This study
GroELΔC_{16} Stop R	5′ ATACCGCCAGCAGCGCCTSAGTCAGCCGCATCGTTTTTCG 3′	This study
GroELΔC_{28} Stop F	5′ ATGATCACCACCGAATGCTAAGTTACCGACCTGCCGAAA 3′	This study
GroELΔC_{28} Stop R	5′ TTTCGGCAGGTCGGTAACTSAGCATTCGGTGGTGATCAT 3′	This study
AAM F	5′ CTAGCGCGGCGATGGCGGCGATGGCGGCGATGGCGGCGATGATGA 3′	This study
AAM R	5′ GCATCATCGCCGCCATCGCCGCCATCGCCGCCATCGCCGCTSCGA 3′	This study
GGD F	5′ CTAGCGGCGGCGATGGCGGCGATGGCGGCGATGGCGGCGATGATA 3′	This study
GGD R	5′ GATCATCGCCGCCATCGCCGCCATCGCCGCCATCGCCGCCTSCGA 3′	This study
PPM F	5′ CTAGCCCGCCGATGCCGCCGATGCCGCCGATGCCGCCGATGATGA 3′	This study
PPM R	5′ GCATCATCGGCGGCATCGGCGGCATCGGCGGCATCGGCGGTTCGA 3′	This study
GLRTF	5′ CCGTCAGCAGATTGAAGAAGC 3′	This study

Supplementary Results and Discussion

Suitability of the Normal Mode Analysis for studying CTS dynamics. As CTSs are glycine rich they are assumed to be extremely flexible and, consistent with this, they have been unresolved in structural studies. Since CTSs assumed distinct conformations in the client-binding tight (*T*) state and the client encapsulating relaxed (*R"*) states, which could be structurally represented as *cis* and *trans* conformations (Fig. 3a), we explored if the CTS transitions between the two conformations correlate with the functional transitions of the chaperonin. To do this, we employed *in silico* methods to explore the path between the two conformational endpoints. Several computational methods, including molecular dynamic simulation tools that were developed to study allostery and dynamics of chaperonins, especially that of GroEL[3-9] proposed successive states and sub-states of the chaperonin cycle, were unable to explore the precise conformational transitions between these states. Moreover, for a mega-dalton system like the GroEL-GroES system with extremely transient multiple high-energy conformational states, obtaining an all-atom resolution of these transition states is computationally unaffordable. Therefore, Normal Mode Analysis has been developed into a tool to study intrinsic collective dynamics of the chaperonins using coarse-grained models[10-12]. Regular improvements in these tools enabled successful dissection of the conformational transitions in GroEL, such as the Brownian dynamics method to study the conformational transitions in GroEL subunit dimers and heptamers[13], adaptive anisotropic Elastic Network Model (ENM)[14] and a combinatorial method to construct coarse-grained models to deliver all-atom accuracy[15-17]. These studies enabled several novel proposals to be made concerning the heterogeneity and succession of the transition pathways. Taking leads from these pioneering methods, we have subjected the tail-

engineered heptameric GroEL models, one in *T* state (*trans* ring) and the other in *R″* (*cis* ring) state, to NMA with ENM using a C-alpha force field (Fig. S6) and selected the global modes (the modes with lowest Eigenvalues) to generate successive states that help us to model the possible structural dynamics in chaperonin action.

To assess the suitability of the method for studying chaperonin dynamics, we checked if the outcome predicts experimentally observed characteristic features of GroEL dynamics and mechanism of action. Results showed (a) a large rotation and consequent characteristic upward displacement (with reference to the equatorial domains) of the client binding helices, H and I of the apical domains (Fig. 3d), (b) a downward movement (~15°) of the helices F and M of intermediate domains (Fig. 3d), which is essential for releasing apical domains into the *R″* state and for stabilizing the ATP binding pocket and (c) an outside-in movement of the apical domain's *wing*, constituted by the hydrophilic helices K and L, that propels the apical domains to move upwards, and thereby increases the volume and hydrophilicity of the cavity. Moreover, the correlation matrices also predicted the following characteristic features of GroEL dynamics, (a) a strong anti-correlated motion between the intermediate domain and the adjacent equatorial and apical domains (Fig. S7b), and (b) a strong positive correlation between adjacent subunits within a ring and a strong negative correlation with the diagonally opposite subunits (Fig. S7b), implying that the adjacent subunits move together in one direction while the diagonally opposite subunits move in opposite direction. In other words, such correlated motion in a ring of odd numbered subunits indicates that all the subunits move closer or farther concurrently as has been observed for GroEL experimentally. Interestingly, higher atomic displacements for four of the seven subunits in *cis* and *trans* rings (Fig. S6), supports the notion that action by four out of seven

subunits of GroEL[18,19] and GroES[20] are sufficient for a functional chaperonin cycle. Furthermore, strong anti-correlated motions were observed between the apical domains of diagonally opposite subunits implying that the apical domains move into and out of the cavity in synchrony during the chaperonin functional cycle (Fig. S7 and S8). The transition path between the *T* and *R”* states encompassed several nucleotide-dependent intermediate states (Fig. 3b and Movie S1), that have also been seen in structural and biochemical studies, suggesting that the observed CTSs fluctuations are biochemically relevant. In addition, the transition map revealed several novel structural features, especially of the CTSs, that could comply with the functional requirements (Fig. 3d and Movie S1). Even though the transition from the *T* to *R”* state is characterized by multiple salt-bridge switching events, since only alpha carbons were considered for NMA, observations are limited to characteristic secondary structural changes in the molecule (Fig. 3). Taken together, the correlation matrices (Fig. S7 and S8), which are consistent with experimental findings on the characteristic cooperativity between the subunits within a ring, further validated the suitability of NMA for GroEL structural studies.

Estimation of the torsion angles, ϕ, ψ and α, and the movement of α carbon atoms, provided further insights into these dynamics. The fluctuations in the torsion angles and the movement of α carbons were calculated for the entire heptamer, at every transition from the *T* state and were averaged for a single subunit (Fig. 3d). Understandably, the majority of the fluctuations and movements were observed in the apical domains and the CTSs (Fig. 3d), suggesting, in combination with the observed perfect anti-correlated motion between these two segments (Fig. 4d), that these two segments move into the cavity while GroEL traverses from the *T* to *R”* state and *vice versa* (Movie S1). The client binding helices, H and I, show fascinating

movements during this transition. Notable changes in the torsion angles before but not within helix H, in combination with the large movement of α carbons (Fig. 3d), directly indicate that this helix, although it traverses ~ 35 Å, moves *en bloc*. On the other hand, though the said changes are similar for helix I and thus move the helix *en bloc*, a decrease in the Φ angle observed at Leu 234 points to a kink introduced within this helix during this transition (Fig. 3d) and therefore suggests that the two client binding helices follow distinct allosteric transitions. The master helix of the intermediate domain, helix M, also shows a similar tendency, but with a significantly smaller movement. Interestingly, although these important helices showed *en bloc* movements while they traverse even large molecular distances, the CTSs, probably owing to their glycine-rich nature, showed very significant movements during the chaperonin cycle, as shown by the large fluctuations in all its torsion angles (Fig. 3d).

Furthermore, as the *T* state CTS is implicated in binding the client proteins via their exposed hydrophobic regions, the conformational transitions of the CTS from the conical *T* to extended *R''* states (Fig. 3) would be likely to unfold the bound 'partially-folded' or 'kinetically-trapped' client proteins when being internalized. Therefore, the close-to-open state transition of CTS is consistent with a model where the CTSs are involved in unfolding of the client proteins prior to their refolding in the cavity. Taken together, these observations establish that the CTSs exhibit large fluctuations that are correlated with the movements of the apical domains and suggest that these two distinct parts of GroEL function in synchrony in client recognition and encapsulation.

Throughout the Normal Mode Analysis, heptameric single-ring models were chosen over the protomer since the CTS in one protomer exhibited unusually large motions that are about

four to ten times stronger than the averaged atomic displacements in the rest of molecule (Fig. S5). Consequently, these fluctuations remained uncorrelated with the rest of the molecule (Fig. S6). On the other hand, 10 ns molecular dynamic simulations of the 13 residue peptide variants, $(GGM)_4M$, $(AAM)_4M$, $(GGD)_4D$ and $(PPM)_4M$, predicted a β-turn like secondary structure for $(GGM)_4M$ peptide (data not shown). Such high atomic displacements and changes in secondary structural features are biochemically unattainable for CTS in its natural abode inside the GroEL cavity, due to spatial and stearic constraints.

Supplementary References

1. Guzman LM, Belin D, Carson MJ, Beckwith J. Tight regulation, modulation, and high-level expression by vectors containing the arabinose PBAD promoter. *J Bacteriol* 1995; **177**(14): 4121-30.
2. Amann E, Ochs B, Abel KJ. Tightly regulated tac promoter vectors useful for the expression of unfused and fused proteins in Escherichia coli. *Gene* 1988; **69**(2): 301-15.
3. Ma J, Karplus M. The allosteric mechanism of the chaperonin GroEL: a dynamic analysis. *Proc Natl Acad Sci U S A* 1998; **95**(15): 8502-7.
4. Ma J, Sigler PB, Xu Z, Karplus M. A dynamic model for the allosteric mechanism of GroEL. *J Mol Biol* 2000; **302**(2): 303-13.
5. Stan G, Thirumalai D, Lorimer GH, Brooks BR. Annealing function of GroEL: structural and bioinformatic analysis. *Biophys Chem* 2003; **100**(1-3): 453-67.
6. Chennubhotla C, Bahar I. Markov propagation of allosteric effects in biomolecular systems: application to GroEL-GroES. *Mol Syst Biol* 2006; **2**: 36.
7. Thirumalai D, Hyeon C. Signalling networks and dynamics of allosteric transitions in bacterial chaperonin GroEL: implications for iterative annealing of misfolded proteins. *Philos Trans R Soc Lond B Biol Sci* 2018; **373**(1749).
8. Horovitz A, Amir A, Danziger O, Kafri G. Phi value analysis of heterogeneity in pathways of allosteric transitions: Evidence for parallel pathways of ATP-induced conformational changes in a GroEL ring. *Proc Natl Acad Sci U S A* 2002; **99**(22): 14095-7.
9. Krebs WG, Gerstein M. The morph server: a standardized system for analyzing and visualizing macromolecular motions in a database framework. *Nucleic Acids Res* 2000; **28**(8): 1665-75.
10. Ma J. Usefulness and limitations of normal mode analysis in modeling dynamics of biomolecular complexes. *Structure* 2005; **13**(3): 373-80.
11. Bahar I, Rader AJ. Coarse-grained normal mode analysis in structural biology. *Curr Opin Struct Biol* 2005; **15**(5): 586-92.
12. Cui Q, Bahar I. Normal mode analysis : theory and applications to biological and chemical systems. Boca Raton, Fla. ; London: Chapman & Hall/CRC; 2006.
13. Hyeon C, Lorimer GH, Thirumalai D. Dynamics of allosteric transitions in GroEL. *Proc Natl Acad Sci U S A* 2006; **103**(50): 18939-44.
14. Yang Z, Majek P, Bahar I. Allosteric transitions of supramolecular systems explored by network models: application to chaperonin GroEL. *PLoS Comput Biol* 2009; **5**(4): e1000360.
15. Na H, Jernigan RL, Song G. Bridging between NMA and Elastic Network Models: Preserving All-Atom Accuracy in Coarse-Grained Models. *PLoS Comput Biol* 2015; **11**(10): e1004542.
16. Na H, Lin TL, Song G. Generalized spring tensor models for protein fluctuation dynamics and conformation changes. *Adv Exp Med Biol* 2014; **805**: 107-35.
17. Na H, Song G. The performance of fine-grained and coarse-grained elastic network models and its dependence on various factors. *Proteins* 2015; **83**(7): 1273-83.
18. Motojima F, Chaudhry C, Fenton WA, Farr GW, Horwich AL. Substrate polypeptide presents a load on the apical domains of the chaperonin GroEL. *Proc Natl Acad Sci U S A* 2004; **101**(42): 15005-12.
19. Chapman E, Farr GW, Fenton WA, Johnson SM, Horwich AL. Requirement for binding multiple ATPs to convert a GroEL ring to the folding-active state. *Proc Natl Acad Sci U S A* 2008; **105**(49): 19205-10.
20. Nojima T, Murayama S, Yoshida M, Motojima F. Determination of the number of active GroES subunits in the fused heptamer GroES required for interactions with GroEL. *J Biol Chem* 2008; **283**(26): 18385-92.

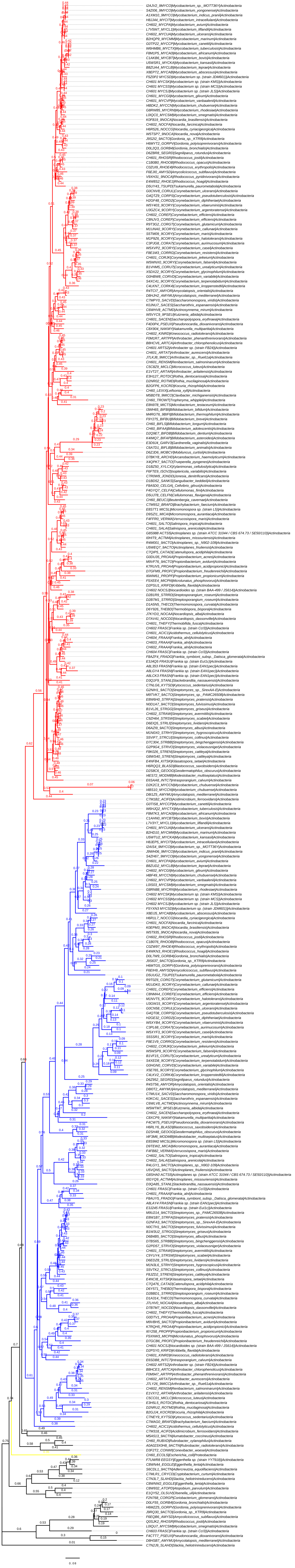

Supplement: Supplementary file 1 — Supplementary information [file 42003_2025_7927_MOESM1_ESM.pdf]
