# Supplementary material for: Genetic and structural insights into the functional importance of the conserved gly-met-rich C-terminal tails in bacterial chaperonins
Source: Commun Biol. 2025 Apr 8;8:555. doi: 10.1038/s42003-025-07927-x (PMC11978752; doi:10.1038/s42003-025-07927-x)
Supplement: Supplementary file 2 — Description of Additional Supplementary Files [file 42003_2025_7927_MOESM2_ESM.pdf]

Description of Additional Supplementary Files

File name: Supplementary data file 1

Description: Excel file containing the metadata of the Carboxy terminal regions of Actinobacterial chaperonins used in this study.

File name: Supplementary data file 2

Description: Excel file presenting a comparison of the overall divergence in the full length and CTS regions.

File name: Supplementary movie 1

Description: Movie showing the structural dynamics of the carboxy terminal regions of chaperonins.
